# Supplementary material for: Chromothripsis during telomere crisis is independent of NHEJ, and consistent with a replicative origin
Source: Genome Res. 2019 May;29(5):737–49. doi: 10.1101/gr.240705.118 (PMC6499312; doi:10.1101/gr.240705.118)
Supplement: Supplemental Material [file supp_gr.240705.118_Supplemental_file_1.zip › contigs/annotated_contigs/DB115/contig.2.DB115_length_638_mean_cov_6.26645768025.docx]

**DB115_length_638_mean_cov_6.26645768025**

```
TAG|CAAACTCCAACAGACCTGCAGCTGAGGGTCCTGACTGTTAGAAGGAAAACTAACAAACAGAAAGGACATCCACACCAAAAACCCA
    >chr10:1272364-1272603 - E=5e-129 p=4e-02
TCTGTACATCACCATCATCAAAGACCAAAGGTAGATAAAAGTACAAAGATGGGGAAAAAAACAGAGCAGAAAAACTGGAAACTCTAAAA

ATCAGAGCACCTCTCCTCCTCCAAAGGAATGGAGTGCCTCACCAGCAATGGAACAAAGCTGGATG|TCTTAAAACATCCAGCTTT|GTG
                                                                                     >c
TCTTAATCACTGTAACGTTATAGTGAGTCTTAAAGTAGGTTCAGAACCTCCAGCTTTGCTGTGGCCCTTGTAAATGTTTTGCATTTTTA
hr10:883142-883519 - E=4e-215
TATGAATTTTAGAATCATCATTACAATTTTTCAAAAAGTCCTGTTGGTGTTTTTATTGGGATTGCATTGATTGTATAAATTCACTCGAG

AAGAATTCACGCATTCTACCAGTGTTAAGTTTTACAACCCACAAGCACAGAATATGTCTTTATGTAGGTCTTTAAAATGTTCTTTTGGC

AGTGTTTTGCAGTTTTCAGCATATGAGTCTTTGATGATCTTTTGTCAGATTAAGATATAAAAACTAAGTTACAGTCATGCACCAGTTAC

CTTTCTTGTAGGGTGAGC
```
